# A Case Report on Neuroleptic Malignant Syndrome (NMS): How to Approach an Early Diagnosis

**DOI:** 10.7759/cureus.23695

**Published:** 2022-03-31

**Authors:** Tanya Paul, Alvina Karam, Trissa Paul, Hanyou Loh, Gerardo F Ferrer

**Affiliations:** 1 Psychiatry, Larkin Community Hospital, South Miami, USA; 2 Medicine, Avalon University School of Medicine, Curacao, CUW; 3 Internal Medicine, Hayatabad Medical Complex Peshawar, Peshawar, PAK; 4 Neurology, Larkin Community Hospital, South Miami, USA; 5 Research, Avalon University School of Medicine, Curacao, CUW; 6 Medicine, National University of Singapore, Singapore, SGP

**Keywords:** quetiapine, antipsychotics, dopamine, schizophrenia, neuroleptic malignant syndrome (nms)

## Abstract

Neuroleptic malignant syndrome (NMS) is a life-threatening neurological emergency that has been observed to occur in some patients following the administration of anti-dopaminergic agents or the rapid withdrawal of dopaminergic medications.

In this report, the authors present a case of a 51-year-old male patient with a known history of cocaine abuse, who was given quetiapine during his hospitalization. This precipitated an episode of NMS that eventually concluded uneventfully due to quick diagnosis and management. Prompt recognition of the condition is required to reduce significant morbidity and mortality. Ultimately, maintaining vigilance for the clinical features of NMS is crucial for timely diagnosis and intervention.

## Introduction

The neuroleptic malignant syndrome is a severe idiosyncratic reaction, commonly occurring in response to the use of potent psychotropic agents. Less commonly, it occurs following the administration of low-potency psychotropic agents or the rapid withdrawal of dopaminergic medications. This is characterized by high fever, altered mental status (AMS), severe muscle rigidity, and signs of autonomic dysfunction. Leukocytosis and elevated serum creatine phosphokinase are also common laboratory findings [1].

The first case of NMS was reported in 1956, following which additional case reports rapidly surfaced. These cases were reported shortly after the introduction of an antipsychotic drug, chlorpromazine, which functions primarily through the blocking of the dopamine-2 (D2) receptor [1]. A study reviewing data of psychiatric inpatients receiving neuroleptics between 1966 and 1997 found that the incidence of NMS ranged from 0.2% to 3.2% [2]. In another study that surveyed patients between 1993 and 2000 under a drug safety program, the incidence of NMS was noted to range from 0.01% to 0.02% [3]. In a report by Otani et al. (1991), it was stated that a Japanese family comprising a mother and two daughters all experienced NMS when placed on neuroleptics [4].

The most obvious risk factor, however, pertains directly to the neuroleptic therapy itself. Oily long-acting depot neuroleptics and rapid titration to achieve desired therapeutic serum levels pose greater risks [5]. Although NMS is considered a neurologic emergency, no evidence-based treatment approach currently exists. Aggressive supportive measures are recommended, which include the immediate cessation of the causative anti-dopaminergic agent or reinstitution of the withdrawn dopaminergic medication, aggressive hydration, and the use of cooling blankets and ice packs to combat the hyperthermia. Metabolic derangements, if present, should also be corrected. In this case report, we discuss a 51-year-old male who developed NMS after being treated with antipsychotics for schizophrenia.

## Case presentation

A 51-year-old Hispanic male with a past psychiatric history of bipolar type 1, hypertension, diabetes type 2, and chronic obstructive pulmonary disease (COPD) presented to us for aggression and bizarre behavior. He had cuts and bruises all over him. He stated that he was a "Christian spy and was going to save his people." The patient also thought we were underwater, and he saw a large blowfish circling him the entire time. He always heard some voices and has been seeing shooting needles and the devil, ever since he could remember. He also complained of severe chest pain on his left side and breathing difficulties. This patient’s known drug allergies included haloperidol, chlorpromazine, and fluphenazine. He used crack cocaine 12 years ago. He consumed marijuana with his food, drank a beer once or twice a day, and returned with a negative response to all of the CAGE (Cut down, Annoyed, Guilty, and Eye-opener) questions.

General physical examination findings were unremarkable. On mental status examination, the patient appeared to be poorly groomed and disheveled. He was guarded and suspicious but cooperative. His speech was loud and then became increasingly monotonous throughout his hospital stay. No abnormal movements were noted at the time. He was able to read but unable to write a complete sentence. He was hyper-alert and only oriented to person but not oriented to place or time. The patient’s mood was preoccupied, and his affect was labile, ranging from worrisome to paranoid to tearful. His thought content consisted of bizarre delusions (grandiose and persecutory), hyper-religiosity, loosening of associations, flight of ideas, and both auditory and visual hallucinations [6-7]. He exhibited tangentiality, disorganized thought process, poor judgment, and insight. His symptoms were consistent with schizophrenia [7].

The patient’s scheduled medications were quetiapine 100 mg orally (PO) every morning and 300 mg PO every night, valproic acid 500 mg twice daily (BID) PO, zolpidem 10 mg PO every night, and lithium carbonate 450 mg BID PO. His medications, taken as needed (PRN), were magnesium hydroxide 30 mL PO every night, diphenhydramine 50 mg every eight hours IM, olanzapine 5 mg every six hours IM, nicotine polacrilex 2 mg every two hours, lorazepam 2 mg PO every six hours, benztropine 2 mg every six hours PO, benztropine 2 mg every six hours IM, and acetaminophen 650 mg PO every four hours.

The patient was combative and was having vivid auditory and visual hallucinations. He was acutely treated with benzodiazepines, which did not sedate him or help with his belligerence. Quetiapine, an atypical antipsychotic, believed to have a lower risk for NMS than typical antipsychotics, was administered.

The patient was transferred to the critical care unit (CCU) because his creatine phosphokinase (CPK) was 8,271 U/L and his creatine kinase myocardial band (CK-MB) was 28.6 IU/L. He was given a single dose of 300 mg Quetiapine before he started deteriorating rapidly. He became profusely diaphoretic, tachycardic, tachypneic, exhibited altered mental status (AMS), increasing CPK levels, and extreme muscle rigidity. A diagnosis of NMS was suspected per the Diagnostic and Statistical Manual of Mental Disorders, Fifth Edition (DSM-5) diagnostic criteria. The patient was treated rigorously with intravenous (IV) fluids, as his CPK continued to rise. His CPK then trended down to 6,661 U/L and his CK-MB normalized to 15.2 IU/L. The patient still had a fever of 101.6 °F, was tachycardic (heart rate of 130bpm), diaphoretic, severely dehydrated, had altered mental status, and his respiratory rate was 30 breaths per minute. He was still on restraints due to his aggression and was also refusing all his medications.

Because this episode occurred right after his exposure to an atypical dopamine antagonist, 300 mg of quetiapine, all future doses of quetiapine were withheld even though the patient was still actively having auditory, visual hallucinations, and persecutory delusions, for fear of inducing another NMS episode or exacerbating his current state. After four days, he had recovered from NMS and was observed to be in a catatonic state.

## Discussion

NMS is seen mostly with the use of high-potency first-generation antipsychotic agents, such as haloperidol and fluphenazine, although cases of NMS have been reported following the use of both low-potency first-generation and second-generation antipsychotic drugs as well. Selective antiemetic drugs, such as metoclopramide, promethazine, and levosulpiride, have also been known to cause NMS [8-9]. In our patient, after a single dose of 300 mg quetiapine, an atypical antipsychotic, signs of NMS were evident.

NMS usually begins with a collection of signs, including muscle cramps and rigidity, labile blood pressure, AMS, hyperpyrexia, and arrhythmias. The muscle rigidity of NMS is postulated to be caused by D2-receptor blockade and may manifest in many ways, including oculogyric crisis and choreiform movements [5]. Velamoor et al. (1994) reviewed 340 clinical reports of NMS available in the literature and discovered that in 70.5% of these cases, patients often progressed in the order of AMS, muscle rigidity, and hyperpyrexia, before finally arriving at autonomic dysfunction. As many as 82.3% of these cases manifested initially with a single presenting sign of AMS or muscle rigidity. The onset of hyperpyrexia may only occur 24 hours following the first onset of signs and/or symptoms [10]. Progression and deterioration, however, often occur rapidly. Hematological and biochemical changes follow, with leukocytosis, elevated serum CK secondary to hyperkinesia and subsequent rhabdomyolysis, as well as metabolic acidosis [5].

Clinically, NMS also needs to be distinguished from its mimics, including serotonin syndrome encephalitis, toxic encephalopathy, and malignant hyperthermia, all of which present with a similar constellation of signs. A carefully taken and detailed drug history is crucial in delineating NMS from other differentials and for appropriate treatment to be started. Once NMS is diagnosed, it is most important to discontinue the offending agent. Sustaining a euvolemic state and bringing CPK levels down to optimal is crucial to care [8]. This may be achieved through the administration of high-volume IV fluids to keep the patient hydrated, decreasing CPK levels, and preventing rhabdomyolysis and hence renal failure [8]. The reason for the severe dehydration commonly seen in NMS patients is due to the excessive amount of fluid being lost by fever and diaphoresis [8].

The majority of cases of NMS resolve in the course of two weeks (the mean is seven to eleven days) [8]. Mortality rates for NMS used to be greater than 30% in the last few decades [11]. Today, the mortality rate is between 5% and 20% [8]. If there has been severe hypoxia or significantly long-standing elevated temperatures, the chances of lifetime neurologic damage are very high [8].

Meanwhile, should the NMS be severe, the empiric administration of bromocriptine mesylate and dantrolene sodium, a dopamine agonist and muscle relaxant, respectively, is recommended to shorten the course of NMS [1]. Increased physician awareness of NMS has led to mortality rates dropping from 30% to 10% over the past few decades. Deaths that occur are often due to disseminated intravascular coagulopathy (DIC), arrhythmias, or failure of the cardiopulmonary or renal systems. Most patients who survive make a complete recovery within two weeks. However, any delay in treatment or diagnosis could result in significant morbidity due to cardiopulmonary or renal complications [1].

## Conclusions

Neuroleptic malignant syndrome is a medical emergency. Since our patient was allergic to most antipsychotics, exhibiting disorganized speech, hallucinations, and bizarre delusions, electroconvulsive therapy was recommended for him. Electroconvulsive therapy is a treatment option for those that cannot tolerate or no longer respond to antipsychotic medications. A substance abuse program should also be considered due to his history of comorbid substance abuse and its link to NMS. Our patient may have tolerated antipsychotics for his schizophrenia without it leading to NMS or a drug allergy if he did not have a history of polysubstance abuse.

Given the rise in psychiatric illness and use of antipsychotics, as well as the incidence of substance abuse in the United States, physicians must be aware of the clinical signs and symptoms of NMS, to arrive at a prompt and accurate diagnosis and begin appropriate treatment in a timely manner.

## References

[1] Berman BD (2011). Neuroleptic malignant syndrome: a review for neurohospitalists. Neurohospitalist.

[2] Pelonero AL, Levenson JL, Pandurangi AK (1998). Neuroleptic malignant syndrome: a review. Psychiatr Serv.

[3] Stübner S, Rustenbeck E, Grohmann R (2004). Severe and uncommon involuntary movement disorders due to psychotropic drugs. Pharmacopsychiatry.

[4] Otani K, Horiuchi M, Kondo T, Kaneko S, Fukushima Y (1991). Is the predisposition to neuroleptic malignant syndrome genetically transmitted?. Br J Psychiatry.

[5] Oruch R, Pryme IF, Engelsen BA, Lund A (2017). Neuroleptic malignant syndrome: an easily overlooked neurologic emergency. Neuropsychiatr Dis Treat.

[6] Marder S, Davis M (2019). Clinical manifestations, differential diagnosis, and initial management of psychosis in adults. UpToDate.

[7] Fischer BA, Buchanan RW (2022). Schizophrenia in adults: clinical manifestations, course, assessment, and diagnosis. UpToDate.

[8] Wijdicks EFM (2019). Neuroleptic malignant syndrome. UpToDate.

[9] Wijdicks EFM (2019). Drugs that can cause neuroleptic malignant syndrome. UpToDate.

[10] Velamoor VR, Norman RM, Caroff SN, Mann SC, Sullivan KA, Antelo RE (1994). Progression of symptoms in neuroleptic malignant syndrome. J Nerv Ment Dis.

[11] Simon LV, Hashmi MF, Callahan AL (2019). Neuroleptic Malignant Syndrome. https://www.ncbi.nlm.nih.gov/books/NBK482282/.

